# "*Tremendous financial burden*": Crowdfunding for organ transplantation costs in Canada

**DOI:** 10.1371/journal.pone.0226686

**Published:** 2019-12-20

**Authors:** Sarah J. Pol, Jeremy Snyder, Samantha J. Anthony

**Affiliations:** 1 The Hospital for Sick Children, Toronto, Ontario, Canada; 2 Faculty of Health Sciences, McMaster University, Hamilton, Ontario, Canada; 3 Faculty of Health Sciences, Simon Fraser University, Burnaby, British Columbia, Canada; 4 Factor-Inwentash Faculty of Social Work, University of Toronto, Toronto, Ontario, Canada; 5 Canadian Donation and Transplantation Research Program, Edmonton, Alberta, Canada; Imperial College Healthcare NHS Trust, UNITED KINGDOM

## Abstract

Online crowdfunding platforms such as GoFundMe are used to raise funds for health-related expenses associated with medical conditions such as organ transplantation. By investigating crowdfunding in Canadian organ transplantation, this study aimed to increase understanding of the motivations and outcomes of organ transplantation crowdfunding. Canadian liver and kidney transplantation campaigns posted to GoFundMe between May 30 & 31 2018 were identified and after exclusion, 258 kidney and 171 liver campaigns were included in study. These campaigns were coded for: worthiness of the campaign recipient, requested financial and non-monetary contributions, how monetary donations would be spent, and comments on the Canadian health system, among others. Results suggest Canadian organ donors, transplant candidates, recipients, and their families and caregivers experience significant financial difficulties not addressed by the public health system. Living and medication costs, transportation and relocation expenses, and income loss were the expenses most commonly highlighted by campaigners. Liver campaigns raised nearly half their goal while kidney campaigns received 11.5% of their requested amount. Findings highlight disease burden and the use of crowdfunding as a response to the extraordinary costs associated with organ transplantation. Although crowdfunding reduces some financial burden, it does not do so equitably and raises ethical concerns.

## Introduction

Medical crowdfunding is a rapidly growing practice where campaigners use online platforms to raise money for health-related needs via social networks. By far the largest charitable crowdfunding platform is GoFundMe, which dominates the medical crowdfunding market in North America and globally. GoFundMe has exhibited rapid growth in the number of campaigns it hosts and money raised, demonstrating widespread and growing interest in medical crowdfunding. Between its launch in 2010 and 2016, GoFundMe raised $930 million (USD) out of a total of $2 billion for health-related costs [1]. During 2017, one third of GoFundMe campaigns were for health-related expenses and this platform raised $650 million for this type of campaign [2].

Medical crowdfunding is used to raise funds both for direct and indirect health-related expenses. Direct expenses can include medical treatment where campaigners lack or have limited insurance coverage and payment for prescriptions, faster access to medical services, access to perceived higher quality services, and unproven or experimental medical treatments. It is also often used for indirect health-related needs such as income support while the campaigner is unable to work due to illness, travel expenses while seeking care, medical equipment and home renovations needed due to illness or disability, and expenses incurred by family members and caregivers [3,4]. While medical crowdfunding is often associated with countries with limited public health insurance coverage like the United States, countries with more robust public insurance systems like Canada and the United Kingdom also see substantial numbers of medical crowdfunding campaigns [5,6].

Among the medical conditions for which campaigners are seeking funding through crowdfunding are health expenses and needs related to solid-organ transplantation. This includes organ transplant candidates and recipients in Canada, where direct medical costs of organ transplantation are largely borne by public health insurance. In many cases, Canadians face significant wait times related to organ transplantation. Data from the 2018 Canadian Organ Replacement Register (CORR) reported that 2,930 transplant procedures were performed in Canada in 2017, leaving 5,333 individuals on the waiting list in need of transplant [7]. More generally, solid-organ transplantation is acknowledged to be among the most resource-intensive medical and surgical procedures for healthcare institutions [8].

Beyond the systemic and direct medical costs, there are additional patient-borne expenses associated with transplantation that burden organ donors, transplant candidates, recipients, and their families and caregivers [8]. Due to the limited number of transplant centres in Canada, the management of chronic organ failure is often coordinated between regions until the time of listing or transplantation when the candidate and their caregiver need to relocate to an area local to the transplant center [8]. Resulting relocation costs (e.g. accommodations local to the hospital) are the responsibility of the candidate, in addition to maintaining payments on their home (e.g. rent/mortgage). Even when not relocating, organ recipients must also pay for travel to transplant centres (e.g. gas, parking) and non-insured medical supplies and medications. Across many organ groups, time spent on the waiting list for a donor and in-hospital recovery post-transplant can be lengthy, and thus accumulative costs can be daunting.

Caregivers may struggle with indirect medical costs as well. To be eligible as a transplant candidate, many programs in Canada require a designated caregiver/support person, who is obliged to be present throughout the transplant trajectory [9]. Thus, the patient’s caregivers are often unable to work, further exacerbating income loss for the collective support network and straining their means to accommodate transplant-related costs. Organ donors also face income-loss because of prolonged employment leave for the transplantation procedure and recovery. For example, approximately one quarter of kidney donors were required to take six or more weeks off work to accommodate the transplant procedure and recovery, rates which are assumed to be higher for organ recipients [10].

Data on crowdfunding trends and the impact of these campaigns is growing but still limited, especially as it pertains to the specific subset of medical crowdfunding related to solid-organ transplantation [11]. Most publications currently assessing the intersection between social media and solid-organ transplantation have been centric to organ solicitation and donor matching [12, 13]. One study by Durand et al. focused on understanding the impact of specific crowdfunding campaign characteristics through quantitative analysis of 850 campaigns from the crowdfunding platform YouCaring [14]. Results of this study highlighted that campaigns written in the third person, with “positive emotional sentiment”, that were longer in length and indicated a higher goal amount were more profitable than campaigns without these qualities [14]. Another general finding that emerged from this analysis was that regardless of the fiscal success of a campaign, even “modest contributions”, meaning donations amounting to $200–$10,000 USD, were noted to improve the donor/recipient’s ability to cope with transplant associated costs [14].

Despite this emerging data, very little is known about the scope, size, aims, and impact of organ crowdfunding initiatives. Organ donors, candidates/recipients, and their families and caregivers experience financial hardship to varying degrees. Characteristics such as the type of organ transplant, the donor/candidate/recipient’s geographic location in relation to the transplant centre, and the length of time spent on the waiting list for organ donation can be factors that aggravate the degree of financial burden experienced [10, 14]. Thus, fine grained and context specific information about this practice is needed.

Durand et al. demonstrated that crowdfunding for organ transplantation creates significant benefits for patients and their families [14]. While few crowdfunding campaigns assessed in this study raised enough to accommodate one year of transplant associated costs, feedback reported that even “modest” amounts were still impactful and appreciated by patients and families [14]. Nonetheless, critics of medical crowdfunding in general have pointed out a range of ethical issues associated with this practice. These include inequitable distribution of the benefits of crowdfunding, a tendency to obscure systemic failings of medical systems, a loss of medical privacy, and pressure to engage in crowdfunding that undercuts patient autonomy [15–17]. Little is known about how crowdfunding related to organ transplantation fits into this existing critical narrative.

In this study we seek to build on the limited understanding of the drivers, outcomes, and ethical dimensions of crowdfunding by persons in need of organ transplants. We do so by looking at crowdfunding in the specific context of Canadians crowdfunding for needs related to kidney and liver transplantation. In doing so, we hope to both add to existing knowledge of crowdfunding for organ transplantation and pave the way for additional studies in specific health system contexts.

## Materials and methods

Between May 30 and 31, 2018 we used GoFundMe’s internal search engine to identify medical crowdfunding campaigns related to kidney and liver transplantation. This was accomplished by conducting searches limited to the ‘medical’ category and using the search terms ‘kidney’ and ‘liver’ each in conjunction with the terms ‘transplant’, ‘transplantation’, ‘donor’, and ‘donation’. At that time, GoFundMe returned search results first for campaigns originating from the same country as that in which the search was conducted. As this study was conducted in Canada, campaigns originating in Canada were returned first by the search engine. For each of these eight searches, the results were recorded until they no longer returned campaigns originating in Canada. These results were then organized by organ group and duplicate campaigns within each category were eliminated, resulting in 640 kidney and 499 liver campaigns. As this study relies exclusively on publicly available information, the Simon Fraser University Research Ethics committee determined the study was exempt from research ethics review.

Text from each campaign was recorded in a shared document and each campaign was then reviewed for inclusion in the study. The inclusion criteria required that the campaign recipient resided in Canada and that the campaign was raising funds related to kidney or liver transplantation, understood as direct and indirect needs prior to, during, and/or following transplantation. Campaigns not meeting these criteria were eliminated, resulting in 258 kidney and 171 liver campaigns (total n = 429). Information on the campaign title, number of donors, number of shares on Facebook, geographical location of the campaigner, funding requested, funding received, and campaign creation date were then recorded for each campaign in a shared spreadsheet (S1 and S2 Datasets; https://docs.google.com/spreadsheets/d/15kUo6NILxaXbbPmmd0TXu785JJ9lPFbd-gINXFW5FWw/edit?usp=sharing).

Included campaigns were then coded using a coding guide developed by the first author. Information coded included whether the campaign recipient was an adult or pediatric patient, the word length of the campaign, language around the recipient’s perceived culpability for needing an organ transplant, language around the recipient’s worthiness for donations, what financial and non-monetary contributions the campaign requested, how any money donated to the recipient would be used, the reason for utilizing crowdfunding, and comments on the Canadian health system. The first author coded all campaigns using this guide. Rigor was ensured by having the second and third authors individually code 5% of the campaigns, with all authors exchanging and discussing these results during two meetings over the course of coding. Disagreements in coding results were discussed and resolved during these meetings and coding standards were refined throughout this process. During the process of coding, the first author also consulted with the other authors when any questions of interpreting the codes and campaigns arose.

## Results

In total, the 429 campaigns (kidney: n = 258; liver: n = 171) that were analyzed requested $11,258,822 CAD (kidney: $8,353,504; liver: $2,905,318). The kidney campaigns were pledged $961,024 (11.5% of requested) while the liver campaigns were pledged $1,427,297 (49.1% of requested). The average donation to a kidney campaign was $98.81, while the average donation to a liver campaign was $109.60. Each kidney campaign received an average of $3,724.90, and on average liver campaigns received $8,346.77.

There were more donors for the liver campaigns compared to the kidney campaigns (liver: n = 13,023; kidney: n = 9,726). Additionally, the liver campaigns received more Facebook shares than the kidney campaigns (liver: n = 77,661; kidney: n = 70,809).

### Patient age group

Most campaigns for both organ types were created for adult patients (kidney: 86.4%, n = 223; liver: 78.4%, n = 134), rather than pediatric patients (kidney: 10.5%, n = 27; liver: 19.9%, n = 34). In a small number of campaigns, the age of the patient was unspecified and could not be inferred from information provided (kidney: 3.1%, n = 8; liver: 1.8%, n = 3). Pediatric campaigns were more successful than their adult counterparts. Pediatric liver campaigns received 60.0% of the requested funds while adult liver campaigns received 42.2%; pediatric kidney campaigns received 37.1% and adult kidney campaigns receiving 10.6% of requested funds.

### Campaign word length

Kidney and liver campaigns had similar numbers of campaigns in each word length range, including campaigns 100 to 249 words in length (kidney: 31.8%, n = 82; liver: 36.8%, n = 63) and 250 to 500 words in length (kidney: 36.0%, n = 93; liver: 33.9%, n = 58). A similar and smaller proportion of kidney and liver campaigns exceeded 500 words in length (kidney: 19.0%, n = 49; liver: 18.1%, n = 31), and fewer campaigns were between 51 and 99 words (kidney: 9.7%, n = 25; liver: 7.6%, n = 13) and below 50 words in length (kidney: 3.5%, n = 9; liver: 3.5%, n = 6).

### Responsibility for needing organ transplantation

Campaigners offered various reasons to justify why the campaign recipient was worthy of donations and typically explained that the campaign recipient’s situation was not their fault but rather a misfortune. The majority of kidney and liver campaigns offered a medical explanation of the campaign recipient’s disease, condition, and/or situation (kidney: 75.6%, n = 195; liver: 57.9%, n = 99). A much smaller number of campaigns used different language to discuss responsibility for these medical conditions. Specifically, few campaigns noted that the need for an organ transplant was not caused by the campaign recipient’s behavior but was caused, for example, by a specific underlying disease (kidney: 2.3%, n = 6; liver: 7.6%, n = 13). A similarly small number of campaigns emphasized that the campaign recipient never engaged in behavior that would have contributed to their current medical situation (kidney: 1.2%, n = 3; liver: 3.5%, n = 6), with some campaigns highlighting that the campaign recipient lives a healthy lifestyle (kidney: 3.5%, n = 9; liver: 1.2%, n = 2), did not consume alcohol in excess (kidney: 1.2%, n = 3; liver: 1.8%, n = 3) or did not acquire Hepatitis C in a socially unacceptable manner (kidney: 0.0%, n = 0; liver: 4.1%, n = 7). The fact that the disease or condition was genetic, and thus not the campaign recipient’s fault was also mentioned by some campaigns (kidney: 5.4%, n = 14; liver: 4.1%, n = 7). One campaign fell into multiple categories and the remaining campaigns did not discuss responsibility for the campaign recipient’s need for transplant (kidney: 23.6%, n = 61; liver: 40.4%, n = 69).

### Recipient’s worthiness

The most common way in which campaigners discussed why the campaign recipient was deserving or worthy of donations was by stressing that the campaign recipient was an ‘amazing person’ who, for example, was ‘caring’, ‘loveable’, or ‘kind’ (kidney: 33.7%, n = 87; liver: 29.8%, n = 51). Other campaigners highlighted that it was uncharacteristic for the recipient to ask for money (kidney: 14.7%, n = 38; liver: 9.4%, n = 16). A small number of campaigns outlined that the recipient had made positive changes in their life to improve their situation (kidney: 0.4%, n = 1; liver: 1.2%, n = 2), that the recipient investigated other ways to raise money (kidney: 5.4%, n = 14; liver: 0.6%, n = 1), or that, if the recipient had alternative means of gaining financial support, they would not be campaigning (kidney: 7.8%, n = 20; liver: 1.8%, n = 3). A few campaigners explained that the campaign was created because family and friends wanted to assist the candidate or recipient (kidney: 2.7%, n = 7; liver: 4.1%, n = 7). Many campaigns did not discuss why the candidate or recipient was worthy or deserving of financial donations (kidney: 55.0%, n = 142; liver: 62.0%, n = 106).

### Recipient’s ‘role’ in family

Many campaigns emphasized the campaign recipient’s role in their family. Most commonly, kidney and liver campaigners emphasized that the campaign recipient was a parent (kidney: 41.5%, n = 107; liver: 21.1%, n = 36). The futures of young recipients within families were often emphasized in terms of having their whole lives ahead of them (kidney: 24.4%, n = 63; liver: 17.0%, n = 29) and recipients were also often described as a partner (kidney: 25.6%, n = 66; liver: 11.7%, n = 20). Discussing the individual as a grandparent (kidney: 4.3%; n = 11; liver: 6.4%, n = 11), or as a breadwinner (kidney: 2.3%, n = 6; liver: 1.2%, n = 2) was done less often.

### Contribution requests

Nearly all kidney and liver campaigns made a specific ask for support (kidney: 97.7%, n = 252; liver: 96.5%, n = 165). Almost all campaigns asked for financial contributions (kidney: 98.4%, n = 248; liver: 100.0%, n = 165). Prayers were the most commonly requested non-monetary contribution (kidney: 7.5%, n = 19; liver: 13.3%, n = 22). Moral support (kidney: 4.4%, n = 11; liver: 0.6%, n = 1), organ solicitation (kidney: 7.9%, n = 20; liver: 2.4%, n = 4), and other requests such as blood donation (kidney: 0.4%, n = 1; liver: 0.6%, n = 1) were also made, but less frequently.

### Campaign objectives

Kidney and liver campaigns described the purposes to which contributions would be directed. Most kidney campaigns requested funding for living expenses for the recipient while liver campaigns were more evenly divided among expenses related to daily living and relocation, as well as transportation expenses for caregivers (Table 1).

**Table 1:** Categories of planned campaigner expenses.

Code	Kidney (n)	Kidney (%)	Liver (n)	Liver (%)
Patient–living expenses (rent, food, etc.)	129	52.0%	53	32.1%
Patient–relocation expenses (travel, rent, etc.)	74	29.8%	56	33.9%
Patient–transportation expenses (parking, gas, etc.)	41	16.5%	52	31.5%
Caregiver expenses (living expenses, transportation expenses, relocation expenses, etc.)	47	19.0%	63	38.2%
Patient–loss of income	54	21.8%	28	17.0%
Patient–medications	45	18.1%	37	22.4%
Patient family expenses (living expenses, transportation expenses, relocation expenses, at-home costs etc.)	34	13.7%	39	23.6%
Patient–other (gym membership, dental expenses, legal fees)	29	11.7%	20	12.1%
Unspecified	29	11.7%	15	9.1%
Donor expenses (living expenses, transportation expenses, relocation expenses, etc.)	25	10.1%	21	12.7%
Patient–full time care/home care/rehabilitation	6	2.4%	12	7.3%
Patient–adjustments to home	5	2.0%	3	1.8%
Research	3	1.2%	0	0.0%
Patient–long term care facilities	0	0.0%	2	1.2%

### Reasons for utilizing crowdfunding

The majority of kidney and liver campaigns characterized organ transplantation as a financial burden, which was the reason why most campaigners turned to crowdfunding (kidney: 85.7%, n = 221; liver: 77.8%, n = 133). The fact that the contributions requested would make life less stressful (kidney: 34.9%, n = 90; liver: 14.0%, n = 24), provide the opportunity to focus on rehabilitation instead of finances (kidney: 30.6%, n = 79; liver: 11.1%, n = 19), and compensate for lost income (kidney: 23.3%, n = 60; liver: 8.2%, n = 14) were all common reasons for utilizing crowdfunding. A few campaigns employed crowdfunding to advertise a fundraiser to raise money for the campaign recipient (kidney: 3.1%, n = 8; liver: 1.2%, n = 2).

### Comments on the Canadian health system

Most campaigns did not mention the Canadian health system (kidney: 84.5%, n = 218; liver: 76.6%, n = 131). Among those that did, the most common type of comment noted that additional costs are not covered by the health system (kidney: 8.9%, n = 23; liver: 14.6%, n = 25). For example, “The drugs aren't [*sic*] covered under government healthcare even though the transplant is and will cost about $5000 per month” (kidney campaign), “The social worker that she spoke with told her she should start fundraising because she must have at least 10,000 for the extra costs. Well so much for free Alberta health care” (kidney campaign), “For those who are not aware of a transplant process, not all costs are covered by Ontario Health Insurance Plan (O.H.I.P.) We need financial aid to cover out of pocket our travel costs, (flights) hospitality (hotel) and some medical expenses (medicines)” (liver campaign). Campaigners also discussed the health system when they commented on private health insurance not providing full coverage for all expenses associated with organ transplantation (kidney: 7.4%, n = 19; liver: 8.2%, n = 14). A few kidney and liver campaigns discussed additional costs not being covered by an organization that might be thought to provide financial support for organ transplant patients (kidney: 0.4%, n = 1; liver: 2.3%, n = 4), not being satisfied with the care they received in the health system (kidney: 0.8%, n = 2; liver: 2.3%, n = 4), and interprovincial health systems (kidney: 0.4%, n = 1; liver: 1.8%, n = 3).

### Campaigner location

Each campaign recorded the geographical location of the individual who created the campaign (https://www.google.com/maps/d/viewer?mid=1YaCfJaf1jmWW3YsrFgjpKa82LkOzoezQ&ll=52.46792071664809%2C-93.8716895&z=3). In some cases, provinces recorded disproportionately many campaigns of one type (e.g., British Columbia, kidney) or the other (e.g., Saskatchewan, liver). Despite having the second largest population in Canada few campaigns originated from the province of Quebec. This may be due to the large Francophone population in that province and domination of English-language campaigns on GoFundMe (Table 2).

**Table 2:** Geographical location of campaign.

Code	Kidney (n)	Kidney (%)	Liver (n)	Liver (%)
Ontario	83	32.2%	54	31.6%
British Columbia	75	29.1%	30	17.5%
Alberta	43	16.7%	25	14.6%
Manitoba	19	7.4%	17	9.9%
Saskatchewan	15	5.8%	17	9.9%
Newfoundland and Labrador	6	2.3%	10	5.8%
New Brunswick	6	2.3%	9	5.3%
Nova Scotia	5	1.9%	3	1.8%
Quebec	3	1.2%	3	1.8%
Prince Edward Island	2	0.8%	3	1.8%
Yukon	1	0.4%	0	0.0%
Northwest Territories	0	0.0%	0	0.0%
Nunavut	0	0.0%	0	0.0%

## Discussion

These findings demonstrate that Canadians in need of kidney and liver transplantation face significant financial challenges not addressed by the public health system. While direct medical costs are generally covered by the public system, these campaigners still struggled with living costs including housing and food, transportation expenses associated with accessing care including parking and gas, relocation expenses associated with accessing care, loss of income prior to and following transplantation, and payment for medications. Other, less common expenses were associated with caregivers, family members, and organ donors or long-term care and rehabilitation needs. These findings help to support and quantify previously acknowledged gaps in the Canadian healthcare system generally and for kidney and liver transplantation recipients specifically [8–10]. Notably, these findings included few instances of direct organ solicitation. This could be evidence that Canadians are adequately able to access organs for transplantation through the existing system or that they generally do not see crowdfunding websites as a suitable venue for such solicitation.

While these campaigners faced significant non-medical expenses that motivated utilizing crowdfunding, few commented on additional costs not being covered by foundations. The David Foster Foundation, for example, is a charity which provides funds extraordinary expenses for pediatric transplant patients in Canada [18]. However, this organization does not provide financial support for prescription medications, medical equipment nor funding for potential donors in living donor transplant assessments [18]. Additionally, campaigners generally did not criticize the Canadian health system for failing to meet these needs. This finding is in line with findings from other studies that show that crowdfunding campaigns tend to focus on the immediate needs of the candidate or recipient rather than the systemic causes of these needs [17, 19]. Crowdfunding campaigners are encouraged by medical crowdfunding websites such as YouCaring to be positive in their campaigns, which may reduce criticism of systemic problems motivating crowdfunding [19]. One concern with this finding is that it encourages the public to view crowdfunding as a solution to the needs of Canadians in need of kidney and liver transplants. That is, by generally ignoring the systemic causes of these needs and demonstrating discrete cases where Canadians are meeting these needs via crowdfunding, donors to these campaigns may be less likely to view the health system as in need of reform.

The proportion of adult to pediatric kidney and liver campaigns follows the wait list size in Canada. In 2018 there were more adult kidney and liver patients on the waitlist than pediatric kidney and liver patients (kidney: adult = 3,123, pediatric = 27; liver: adult = 507, pediatric = 20) [20]. While some of these campaigners were able to meet their crowdfunding goals, the majority were not. There was also a significant disparity between the two groups studied, with nearly half of those in need of liver transplants meeting their goals compared to only 11.5% of kidney campaigns. This result was in part due to kidney campaigns requesting more money on average than liver campaigns, but liver campaigns also saw more donors per campaign, more money from each donor, and more money received per campaign. This finding mirrors that of Durrand et al., who found liver campaigns receive more on average than kidney campaigns [14].

While we are not certain as to the reason for this disparity, it does highlight a general ethical concern with inequity in crowdfunding. Whereas systems for distributing organs may be driven by values such as efficient and fair allocation, crowdfunding is thought to distribute funding according to norms such as popularity and personal sympathy [16, 17]. Moreover, there is also risk of bias in how crowdfunding campaigns are received by viewers by creating potential advantages for those who are photogenic, sympathetic, or equipped with superior writing skills [14]. This was seen in the campaigns discussed in this study in that campaign recipients were painted as morally worthy of donation and children received a larger percentage of their requests than adults. Campaigners clearly felt pressure to present patients as victims of bad medical luck rather than their own behaviors and generous pillars of their communities who deserved help in return. This, of course, problematically presumes that some individuals are more worthy of medical care than others, violating norms of treating medical care as an entitlement and human right. If access to organ transplantation is understood as an entitlement and matter of justice, then it is ethically problematic to shift to a system of distribution that awards individuals according to perceived moral worthiness or socioeconomic advantage rather than need or achieving good outcomes.

In presenting their cases for donation, these individuals also faced significant costs in terms of their personal and medical privacy [14]. These details included what kind of organ transplant they needed, their status in the process of receiving a new organ, the underlying cause of the need, and regular updates on their health including photographic documentation and expressions of gratitude. As these campaigns tended to focus primarily on living costs, personal financial details including employment status, housing costs, savings, and debt levels were often disclosed. While these disclosures were in a sense voluntary in that crowdfunding itself and the disclosure of personal details are not required of individuals, financial need may have left many campaigners with no other acceptable options for meeting their needs. Similarly, campaigners are frequently advised by medical crowdfunding websites to present these details in order to promote giving and to establish the legitimacy of their campaigns [15, 16, 19]. Moreover, these campaigns often included references to family and caregivers, including minors, whose privacy was undermined by these campaigns. Importantly, this research shows that personal medical and family information is often revealed in order to establish the legitimacy of the campaign and worthiness of the individual for medical care. In addition to problematically reinforcing the distribution of medical resources according to perceived moral worthiness, this aspect of medical crowdfunding can trade on problematic norms of worthiness including stigmatizing those in need of organ transplantation due to stigmatized behaviors such as substance abuse. This loss of privacy raises significant ethical concerns in that a right to personal and medical privacy is often seen as a basic human right. Trading this right for accessing other human rights such as medical treatment and bodily integrity indicates a failure of a society to protect basic human rights. Notably, patients and families do receive advice from healthcare professionals regarding privacy issues when using social media. Henderson et al. also caution patients about posting sensitive information to social media and recommend that hospitals highlight loss of privacy and confidentiality as a psychosocial and psychological risk associated with social media [12].

These findings should prove useful to academics, policy makers, and healthcare providers seeking to better understand and respond to the context of Canadians in need of kidney and liver transplantation. These findings highlight the burden of disease, including provincial differences in the needs of different organ transplant recipients, donors, and family members. Crowdfunding is a response to the extraordinary costs associated with organ transplantation and system gaps that leave many people struggling to meet their living expenses and other non-medical needs prior to and following organ transplantation. This study demonstrates that the Canadian health system includes clear gaps that are being filled by medical crowdfunding. Most clearly, indirect health costs such as relocation expenses, travel, loss of income, and caregiver expenses are burdening many Canadians. While crowdfunding can help to address some of these needs, it does so inequitably and at significant cost to personal privacy. Thus, there is a clear need to systemically address these gaps through policy changes rather than leaving it to the ad hoc responses of crowdfunders and their donors.

Additional research is needed to understand why campaigns for persons undergoing liver transplants are significantly more successful than those related to kidney transplants. This research could include additional studies of Canadian crowdfunding campaigns, including interviews with these campaigners. Study should be given to other national contexts as well in order to determine if the disparities observed here are distinctive to Canada or are more widespread. Such research will also reveal whether the needs of Canadians seeking organ transplants are similar to those of non-Canadians in different health systems.

## Supporting information

S1 Dataset Liver: Shared spreadsheet of liver campaign data. (PDF)

S2 Dataset Kidney: Shared spreadsheet of kidney campaign data. (PDF)

## Abbreviations

USD: United States Dollars
CORR: Canadian Organ Replacement Register
OHIP: Ontario Health Insurance Plan
