# Correction: "*Tremendous financial burden*": Crowdfunding for organ transplantation costs in Canada

**DOI:** 10.1371/journal.pone.0230590

**Published:** 2020-03-12

**Authors:** Sarah J. Pol, Jeremy Snyder, Samantha J. Anthony

The second author, Jeremy Snyder, should also be noted as a corresponding author. Jeremy Snyder’s email address is: jcs12@sfu.ca.
