# Supplementary material for: "Tremendous financial burden": Crowdfunding for organ transplantation costs in Canada
Source: PLoS One. 2019 Dec 20;14(12):e0226686. doi: 10.1371/journal.pone.0226686 (PMC6924656; doi:10.1371/journal.pone.0226686)
Supplement: S1 Dataset — (PDF) [file pone.0226686.s001.pdf]

Title	URL
Deb's Journey with Liver Transplant	https://www.gofundme.com/27ucxx9g
Liver Transplant, For Allexis	https://www.gofundme.com/fcmk3c
Live Liver Transplant	https://www.gofundme.com/live-liver-transplant
Pat is waiting for Liver Transplant	https://www.gofundme.com/pat-is-waiting-for-liver-transplant
Saving Karen Liver Transplant	https://www.gofundme.com/Saving-Karen
Torri's & Kyla's Liver Transplant	https://www.gofundme.com/torri-kylas-liver-transplant
Ronni's Liver Transplant Recovery	https://www.gofundme.com/ronnidixie
Karen's Liver Transplant	https://www.gofundme.com/karens-liver-transplant
Barry Willford's Liver Transplant	https://www.gofundme.com/barrys-liver-transplant-fund
Marion's Liver Transplant	https://www.gofundme.com/marion-brown-liver-transplant
Rhoda's Liver Transplant Recovery	https://www.gofundme.com/z7pf2t8
Mike needs a liver transplant	https://www.gofundme.com/25xrhmw4
Bob's Liver transplant	https://www.gofundme.com/bobs-liver-transplant
Baby Nash Needs A Liver Transplant	https://www.gofundme.com/qggk8eec
Erica's Liver Transplant	https://www.gofundme.com/ericas-liver-transplant
Gabriela's Liver Transplant	https://www.gofundme.com/2mehvyc
Natalie Williams - Liver Transplant	https://www.gofundme.com/natalies-liver-transplant
Sandy's liver transplant journey	https://www.gofundme.com/p-8hzkh
Megan Fraser liver transplant	https://www.gofundme.com/2rp9k3w
Jennifer's liver transplant fund	https://www.gofundme.com/8g2tuvkuw
Douglas McD Liver Transplant	https://www.gofundme.com/dougs-liver-transplant
Chris Bordush's Liver Transplant	https://www.gofundme.com/chrisbordush
Glen Leeman Liver Transplant	https://www.gofundme.com/glen-leeman-liver-transplant
Samara's Fund for Liver Transplant	https://www.gofundme.com/tft8gg
Chantal's liver transplant	https://www.gofundme.com/he39qwzw
Mikylah's Liver Transplant	https://www.gofundme.com/mikylah
Pat Penton's liver transplant	https://www.gofundme.com/sk5gsgfk
Courtney's Liver Transplant Fund	https://www.gofundme.com/radnak
Andrew Brunton's Liver Transplant	https://www.gofundme.com/Andrewbrunton
Cathy's Liver Transplant Fund	https://www.gofundme.com/Cathysliverfund
Pastor Arnold's Liver Transplant	https://www.gofundme.com/pastor-arnolds-liver-transplant
Jasmine's Liver Transplant Fund	https://www.gofundme.com/KleiboerFamily
Terry's Liver Transplant	https://www.gofundme.com/terrys-liver-transplant
Farrah's liver transplant	https://www.gofundme.com/farrahs-liver-transplant
Make Robyn's Liver Transplant happen	https://www.gofundme.com/5g3xc6w
Liver transplant assessment	https://www.gofundme.com/liver-transplant-assessment
LIVER TRANSPLANT N.B. Canada	https://www.gofundme.com/58s8b3s
Mike's fight for a liver transplant	https://www.gofundme.com/mikes-fight-for-a-liver-transplant
Liver Transplant Cancer Conflict	https://www.gofundme.com/liver-transplant-cancer-conflict
Braedons story	https://www.gofundme.com/braedons-story
Greta's battle against cancer	https://www.gofundme.com/gretasguardians
Sweet Tala's Journey To Health	https://www.gofundme.com/3m63mqg
Princess Ava's Broken Heart Fund	https://www.gofundme.com/dvuqazb8
Hope for Jackson	https://www.gofundme.com/jn5uy-hope-for-jackson
New liver for Don (Donald Ruest)	https://www.gofundme.com/liverfordon
Help with Wayne kosher medical	https://www.gofundme.com/help-with-wayne-kosher-medical
Rally Around the Rievajs	https://www.gofundme.com/rally-around-the-rievajs
Eric's Family Fund	https://www.gofundme.com/Whitbread
Roy Howe's health	https://www.gofundme.com/roy-howes-health
Trina's Liver Journey	https://www.gofundme.com/trinasliverjourney
Jacob's Journey	https://www.gofundme.com/kp-jacobs-journey
McKenzie's Fund	https://www.gofundme.com/mckenzies-fund
A helping hand for Dan Michie	https://www.gofundme.com/2c43njpu
Support for Tatum	https://www.gofundme.com/support-for-tatum
HELP A FRIEND AND HIS FAMILY.	https://www.gofundme.com/helpseanfindadonor
Let's Get Rob a Liver!	https://www.gofundme.com/lori-and-robs-liver-troubles
Baby Keaton's Transplant Expenses	https://www.gofundme.com/baby-keatons-transplant-expenses
Roisin's Transplant Surgery	https://www.gofundme.com/roisins-transplant-surgery
A new liver for Kari	https://www.gofundme.com/a-new-liver-for-kari
Support Kim while she saves Scott!	https://www.gofundme.com/SupportKimSavingScott
Help Rafael Beat Cancer	https://www.gofundme.com/397ey-help-rafael-beat-cancer
Kyle's Medical Treatment Fund	https://www.gofundme.com/kg0v0o
A New Liver for Kristopher	https://www.gofundme.com/2k54tq5g
Tkatchuk Family needs our Help!	https://www.gofundme.com/rf4bgk
Colleen's Cure	https://www.gofundme.com/colleens-cure
Shirley Needs a New Liver	https://www.gofundme.com/shirley-needs-a-new-liver
Help save Jillian's life	https://www.gofundme.com/vf7qdf44
Support for Bev Hill's Recovery	https://www.gofundme.com/support-for-bev-hills-recovery
Help Granny Jo in her recovery	https://www.gofundme.com/help-granny-jo-in-her-recovery
Help Delilah Saunders' legal fund	https://www.gofundme.com/help-delilah-saunders
Melissa Davies transplant fund	https://www.gofundme.com/melissadavies
Carries Miracle	https://www.gofundme.com/zuzn4-carries-miracle
Osterlund Family	https://www.gofundme.com/Tanya-Brian
Keith Badura ~ Husband♥Father♥Papa♥	https://www.gofundme.com/2hy4nrw
KRISTA'S 2ND BATTLE WITH CANCER -	https://www.gofundme.com/z9rh9g
Living with Liver Disease	https://www.gofundme.com/living-with-liver-disease
Ethan's Getting A Liver	https://www.gofundme.com/ethans-getting-a-liver
Korey Cotton's Medical Fund	https://www.gofundme.com/korey-cottons-medical-fund
HelpAlexa	https://www.gofundme.com/prayforheralexa
Funeral/Living Expenses	https://www.gofundme.com/5c18i4w
Fight with Karen!	https://www.gofundme.com/fight-with-karen
Support for The Robicheau Family	https://www.gofundme.com/the-robicheau-family
Help My Son /Autoimmune Hepatitis	https://www.gofundme.com/kristianautoimmunehepatitis
Todd Butler Recovery Fund	https://www.gofundme.com/s44m5km5
Support for Katie (aka Superwoman)	https://www.gofundme.com/support-for-katie-aka-superwoman
Help Lindsay's Fight Against Cancer	https://www.gofundme.com/help-lindsays-fight-against-cancer
Carol's Journey to Health	https://www.gofundme.com/j7xetp5f
Help Norma on her Road to Recovery	https://www.gofundme.com/HelpNormaRoadtoRecovery
Clear Dylan's truck loan	https://www.gofundme.com/dylans-truck-loan
Cyrstal Witty (Priest), Liver Trans	https://www.gofundme.com/crystalpriest
Shelby Miller needs our help!	https://www.gofundme.com/4z2q3o
Support Sergio's Road to Recovery	https://www.gofundme.com/simonetti
Bring Kaleigh Home For Christmas	https://www.gofundme.com/hg23oc
Get busy living with a new liver !	https://www.gofundme.com/getbusylivingCindy
Davids medical fund	https://www.gofundme.com/tbdr8-davids-medical-fund
Jody's Time to Survive and Thrive!!	https://www.gofundme.com/Jodyjody-s-time-to-survive-and-thrive
Help For Baby Dax And Family!	https://www.gofundme.com/k7y36wek
Team Sweeney #adamwillbegreatagain	https://www.gofundme.com/team-sweeney-adamwillbegreatagain
Join the Fight Against PSC	https://www.gofundme.com/join-the-fight-against-psc
Tammy's Healing & Recovery Fund	https://www.gofundme.com/tammys-healing-recovery-fund
The gift of teeth for my stepdad	https://www.gofundme.com/the-gift-of-teeth-for-my-stepdad
Help Becca's Health	https://www.gofundme.com/help-beccas-health
Let's help Gary Leigh	https://www.gofundme.com/lets-help-gary-leigh
Mccubbing Family Medical Fund	https://www.gofundme.com/b7uht5pu
Help Support Roy's Family	https://www.gofundme.com/wfpfj8r
Join James' Journey	https://www.gofundme.com/join-james-journey
Val Chandler's Transplant Fund	https://www.gofundme.com/valeries-transplant-fundraiser
A Day For Diane	https://www.gofundme.com/2po8r3w
Family In Need	https://www.gofundme.com/5167p4o
A Mother's Christmas Wish	https://www.gofundme.com/a-mothers-christmas-wish
Help me, help my mom	https://www.gofundme.com/mqvaup-help-me-help-my-mom
I need Your Help !!!!!!!!!!!!	https://www.gofundme.com/Cirrhosis
Support Susie	https://www.gofundme.com/kss434-support-susie
Richard's Journey	https://www.gofundme.com/ep2r24pg
Fundraiser for Gilbert Ball	https://www.gofundme.com/supportgib
Help To Support Dean And Holly	https://www.gofundme.com/437nscw
BELIEVE' in Cindy's Liver Journey	https://www.gofundme.com/039believe039-in-cindy039s-liver-journey
Chantelle McDermott	https://www.gofundme.com/chantelle-mcdermott
Jennifer Specht's Medical Fund	https://www.gofundme.com/jennifer-spechts-medical-fund
Andrew Thomas' Medical Fund	https://www.gofundme.com/AndrewThomas
The Weimar family	https://www.gofundme.com/t7fahk
Dante Rinaldi Medical Relief Fund	https://www.gofundme.com/8mazw8-dante-rinaldi-medical-relief-fund
Nino's Transplant Recovery Fund	https://www.gofundme.com/ninos-transplant-recovery-fund
Prayers and Hope for Ella-Marie	https://www.gofundme.com/yggc44d4
Gerrys medical expenses	https://www.gofundme.com/gerrys-medical-expenses
Hopes,Dreams, Special Little Things	https://www.gofundme.com/hopesdreams-special-little-things
Linda Simard's Transplant Fund	https://www.gofundme.com/linda-simards-transplant-list-appt
New Liver New LIFE	https://www.gofundme.com/la45ss
Doms Dimes	https://www.gofundme.com/doms-dimes
Medical expenses	https://www.gofundme.com/5zb636-35
Nicky's Care/ Fund	https://www.gofundme.com/Nickyslife

Title	URL
Donald's Medical Fund	https://www.gofundme.com/uk39d-donalds-medical-fund
Fundraiser For Richard Maracle	https://www.gofundme.com/fundraiser-for-richard-maracle
Medication Support	https://www.gofundme.com/5gih0wg
help Kandy-Lee Ferris	https://www.gofundme.com/help-for-kandy
Fundraiser for Camilo Revonte	https://www.gofundme.com/gn5e73-i-need-a-liver-replacement
Help Cover Medical Exenses	https://www.gofundme.com/help-cover-medical-exenses
Help support Delilah Saunders	https://www.gofundme.com/help-support-delilah-saunders
my little boys medical bills please	https://www.gofundme.com/my-little-boys-medical-bills-please
My 7 year old nephew needs liver	https://www.gofundme.com/my-7-year-old-nephew-needs-liver
help my brother	https://www.gofundme.com/w8vcs5-help-my-brother
My mom medical bills	https://www.gofundme.com/q4q9h8-my-mom-medical-bills
Francine Gagnon Needs New Liver	https://www.gofundme.com/francine-gagnon-needs-new-liver
Baby Bel Liver Donor Travel cost	https://www.gofundme.com/baby-bel-liver-donor-travel-cost
Please support Mary's journey	https://www.gofundme.com/please-support-marys-journey
Clarence's Medical Fund	https://www.gofundme.com/5agjd-clarences-medical-fund
Kim Clayton's Recovery Fund	https://www.gofundme.com/q84rjv-kims-recovery-fund
Ernie's Double Transplant Surgery	https://www.gofundme.com/2hc59hbg
Kelly & Judy's Transplant Recovery	https://www.gofundme.com/n36qu5nw
Tim Martin transplant support	https://www.gofundme.com/8pktj3-tim-martin-transplant-support
Darren Smith	https://www.gofundme.com/darren-smith
Dino Bluecoat	https://www.gofundme.com/dino-bluecoat
Rebekah's Journey	https://www.gofundme.com/Rebekah-s-Journey
Mark's Medical Fund	https://www.gofundme.com/7d3ppp-marks-medical-fund
Help for end-of-life expenses	https://www.gofundme.com/loveforKourtney
Helping our friend, Brad Bealey	https://www.gofundme.com/helping-a-true-friend-in-need
medical expenses - Randolph gvb	https://www.gofundme.com/medical-expenses-randolph-gvb
Michele and Gary Dycks Support	https://www.gofundme.com/michele-and-gary-dycks-support
Mike's Road to Recovery	https://www.gofundme.com/miketetford
Grace's Gift	https://www.gofundme.com/22xeee4
Elliotts Cancer Fight	https://www.gofundme.com/3tw4jco
Medications & Household Bills	https://www.gofundme.com/ci6r0s
Jens health and care	https://www.gofundme.com/jgehkq3w
ZAYDEN EAGLEPLUME	https://www.gofundme.com/Zayden
Help Baby Sophia Recover!!!	https://www.gofundme.com/kstfuo
Help Save Our Sister Fund	https://www.gofundme.com/yq7f97y
Need help for transportion	https://www.gofundme.com/need-help-for-transportion
Sick Mother Needs Flight for Live Donor	https://www.gofundme.com/bhuu08
Liver Giver Fundraiser	https://www.gofundme.com/amberalain
Coretta's Medical Fund	https://www.gofundme.com/eg3waexs
Love for The Love's	https://www.gofundme.com/khnmpw
