# Supplementary material for: "Tremendous financial burden": Crowdfunding for organ transplantation costs in Canada
Source: PLoS One. 2019 Dec 20;14(12):e0226686. doi: 10.1371/journal.pone.0226686 (PMC6924656; doi:10.1371/journal.pone.0226686)
Supplement: S2 Dataset — (PDF) [file pone.0226686.s002.pdf]

Title	URL
It's time to end the agony!	https://www.gofundme.com/EndTheAgony
Raising Awareness of Kidney Health	https://www.gofundme.com/DancingwRejection
Johns Medical Trips	https://www.gofundme.com/johns-medical-trips
I need a kidney to live	https://www.gofundme.com/bzghzuus
Michael Delorme Medical Expenses	https://www.gofundme.com/michael-delorme-medical-expenses
Fight with Karen!	https://www.gofundme.com/fight-with-karen
Jason Laframboise trying to breathe!	https://www.gofundme.com/otk7po
Support baby Caribou/Sinclair	https://www.gofundme.com/support-baby-caribousinclair
Derek's Kidney Transplant	https://www.gofundme.com/dereks-chronic-kidney-restoration
Matt Hassen's Kidney Transplant	https://www.gofundme.com/matt-hassens-kidney-transplant
Kidney transplant for Ryan Harkness	https://www.gofundme.com/kidney-transplant-for-ryan-harkness
Enrico's Kidney Transplant	https://www.gofundme.com/enrico039s-kidney-transplant
Kidney Transplant Medications	https://www.gofundme.com/kidney-transplant-medications
Kidney transplant	https://www.gofundme.com/eemd2y3d
Kidney transplant family	https://www.gofundme.com/kidney-transplant-family
Darren's Kidney Transplant	https://www.gofundme.com/o85f7k
Claudia's Kidney Transplant Fund	https://www.gofundme.com/claudias-kidney-transplant-fund
Doug ~ No Kidneying Around	https://www.gofundme.com/no-kidneying-kidney-transplant
Sarah's kidney transplant fund	https://www.gofundme.com/sarahs-kidney-transplant-fund
Gladys Njambi Kidney Transplant	https://www.gofundme.com/gladys-njambi-kidney-transplant
Kidney Transplant	https://www.gofundme.com/8gfds-kidney-transplant
Tori Anger Kidney Transplant	https://www.gofundme.com/tori-anger-kidney-transplant-2tudhdg
Mom got call for Kidney Transplant	https://www.gofundme.com/mom-got-called-for-transplant
Kidney Transplant	https://www.gofundme.com/single-dad-with-kidney-disease
Kidney Transplant Expense Fund	https://www.gofundme.com/alonzopowerkidneytransplant
Ron's kidney transplant	https://www.gofundme.com/rons-kidney-transplant
Simeon's Kidney Transplant	https://www.gofundme.com/simeons-kidney-transpant
expenses during kidney transplant	https://www.gofundme.com/expenses-during-kidney-transplant
Support Kassie's kidney transplant	https://www.gofundme.com/2meqdaz8
Jodie Wolf's Kidney transplant fund	https://www.gofundme.com/s5rjkg6c
Help Jason w/ Medical Transport	https://www.gofundme.com/help-jason-get-a-kidney-transplant
Kidney Transplant Fund	https://www.gofundme.com/n9muqk-kidney-transplant-fund
Jenn and Tiana's Kidney Transplant	https://www.gofundme.com/JennTianaKidney
Funds for kidney transplant	https://www.gofundme.com/5j8yxp-funds-for-kidney-transplant
Angeleah's kidney transplant	https://www.gofundme.com/angeleahs-kidney-transplant
Bobby Bates kidney transplant	https://www.gofundme.com/gvmtvs
Brother/Sis live Kidney Transplant	https://www.gofundme.com/Bro-s-Kidney-Trans
Lonnie's Kidney Transplant	https://www.gofundme.com/2hwanyvc
Help for Kidney transplant expenses	https://www.gofundme.com/tgcb3uc
Support Darren's Kidney Transplant	https://www.gofundme.com/nigqzc
Kidney Transplant	https://www.gofundme.com/aherm4-kidney-transplant
Kidney transplant - travel expenses	https://www.gofundme.com/6m8tq8
Owen's Kidney Transplant 5/10/18!	https://www.gofundme.com/owens-kidney-transplant-51018
Murray's Kidney Transplant Recovery	https://www.gofundme.com/zg9w6x6x
jacobs kidney transplant Fundraiser	https://www.gofundme.com/2eawaht4
Tara's Kidney Transplant	https://www.gofundme.com/tara039s-kidney-transplant
HELP ROXANNE KIDNEY TRANSPLANT	https://www.gofundme.com/help-roxanne-kidney-transplant
Sheldons Kidney Transplant Fund	https://www.gofundme.com/gjigyc
Our Kidney Transplant Journey	https://www.gofundme.com/p32cq4
My sister had a kidney transplant	https://www.gofundme.com/my-sister-had-a-kidney-transplant
Hilborn family kidney transplant	https://www.gofundme.com/hilborn-family-kidney-transplant
Medical kidney transplant surgery	https://www.gofundme.com/medical-kidney-transplant-surgery
Kidney transplant	https://www.gofundme.com/vsnx2-kidney-transplant
Kidney Transplant	https://www.gofundme.com/w6u5ev-kidney-transplant
Kidney Transplant For my Friend	https://www.gofundme.com/cmtpr5-kidney-transplant-for-my-friend
Kidney transplant & medical costs	https://www.gofundme.com/ba8tr7-kidney-transplant-medical-costs
Father kidney transplant	https://www.gofundme.com/father-kidney-transplant
RD's Kidney Transplant Expenses	https://www.gofundme.com/rds-kidney-transplant-expenses
Kidney Transplant Struggle flooded	https://www.gofundme.com/377xa1c
Lee's Fight	https://www.gofundme.com/lees-fight
My Sister's Disease	https://www.gofundme.com/My-Sisters-Disease
The Giunta Family Emergency Fund	https://www.gofundme.com/7au70s
A new life for Michael	https://www.gofundme.com/a-new-life-for-michael
Move Mountains For Amanda	https://www.gofundme.com/5baiw2o
Kelly 's Double Organ Transplant	https://www.gofundme.com/HaveaHeartforKelly
Pray for Karris and Kayden.	https://www.gofundme.com/pray-for-kayden-and-karris
Care for Katie	https://www.gofundme.com/w3gfyg-care-for-katie
Get Jes A Kidney & Get Him Walking	https://www.gofundme.com/2j5uass
Support our living 'angel' donor	https://www.gofundme.com/support-our-living-039angel039-donor
Sending LOVE to Olivia the WARRIOR	https://www.gofundme.com/sending-love-to-olivia
Nicki's Kidney Fundraiser	https://www.gofundme.com/nickis-kidney-fundraiser
Sergio Osorio Kidney fund	https://www.gofundme.com/sergio-osorio-kidney-fund
Penn's Kidney Operation	https://www.gofundme.com/pennskidneyoperation
Mike Walsh Transplant Expenses	https://www.gofundme.com/mike-walsh-transplant-expenses
Bergmans for Bruno	https://www.gofundme.com/brucebergman
Endang's Battle with Renal Failure	https://www.gofundme.com/endang039s-battle-with-renal-failure
Ian Daryll's Kidney Recovery Fund	https://www.gofundme.com/ian-darylls-kidney-recovery-fund-2uakg6rk
Operation Kidney	https://www.gofundme.com/shanerobertson
Taylum's Medical Bills	https://www.gofundme.com/es8ji0
MICHELLE NEEDS A KIDNEY	https://www.gofundme.com/michelle-needs-a-kidney
Mike's Kidney Klub.	https://www.gofundme.com/Mikeskidneyklub
For a Teacher with Kidney Disease	https://www.gofundme.com/ongkar
Support for our girl Stephanie	https://www.gofundme.com/support-for-our-girl-stephanie
Tina's Transplant Fund	https://www.gofundme.com/tinas-transplant-fund
Please Help Our Dad, Kevin	https://www.gofundme.com/2h4j4zvw
Kidney for Darrin Crosby	https://www.gofundme.com/kidney-for-darrin-crosby
Al's Kidney Recovery Fund	https://www.gofundme.com/als-kidney-recovery-fund
Support our Friends Nigel&Rhiannon	https://www.gofundme.com/2jessqu4
The Punga's Transplant Fund	https://www.gofundme.com/rodney-beth-need-our-help-2twhvfw
Thank You for your help!	https://www.gofundme.com/cd4nnbjs
Jamie and Charlene need help	https://www.gofundme.com/2gf3a3pg
Funds for Daniel's Support Team	https://www.gofundme.com/coolkid
Dan & Sandi Fleet Family Fundraiser	https://www.gofundme.com/rku7q39g
Irene's Kidney Donation Fund	https://www.gofundme.com/irenes-kidney-donation-fund
Rob's Surgery Towards a new Kidney!	https://www.gofundme.com/robs-reconstruction
Support for Sean and Megan Quinn	https://www.gofundme.com/support-for-sean-and-megan-quinn
Merry Christmas Catherine & Cameron	https://www.gofundme.com/merry-christmas-catherine-cameron
Young Girl Awaiting Transplant	https://www.gofundme.com/j4al70
HELP ME KICK B CELL LYMPHOMA'S ASS	https://www.gofundme.com/2ff9dp-help-me-kick-b-cell-lymphomas-ass
Shandra MacNeill Recovery Fund	https://www.gofundme.com/shandramacneill
Zac Morgan's Kidney Fund	https://www.gofundme.com/2e43ftk5
Lynda's New Kidney - post-op fund	https://www.gofundme.com/healthykidney
"Help Lisa fight the battle"	https://www.gofundme.com/2uhj4-help-lisa-fight-the-battle
Braun family transplant fund	https://www.gofundme.com/svhn88u4
Deedee's fight for life	https://www.gofundme.com/29pxb6ng
Medical Travel expenses	https://www.gofundme.com/5zsmc-medical-travel-expenses
Kidney failure medical expenses	https://www.gofundme.com/zrmtt-kidney-failure-medical-expenses
Steve Crane	https://www.gofundme.com/rg8ce6g
Local couple need life saving help!	https://www.gofundme.com/ournewkidney
Baby Bentley	https://www.gofundme.com/d4n9nr78
Louise Cousins Show Your Love	https://www.gofundme.com/louise-cousins-medical-expences
Rooting for Ryley Bustamante Heck	https://www.gofundme.com/rooting-for-ryley
Dayna needs a kidney and our help!	https://www.gofundme.com/dayna-needs-our-help
Miguel's Donor Fund	https://www.gofundme.com/donorfund
Kurtis Twinn's Get Well Fund	https://www.gofundme.com/kkxoig
Christopher's New Kidney	https://www.gofundme.com/ua28ymk
HELP MY HERO!	https://www.gofundme.com/s2jvu7c
Jeff & Debbie's Transplant Journey	https://www.gofundme.com/jeff-amp-debbie039s-transplant-journey
Help for the David Killacky family	https://www.gofundme.com/help-for-the-david-killacky-family
New Kidney for Hazel	https://www.gofundme.com/kidneyforhazel
Kendra's Medical Funds.	https://www.gofundme.com/kendras-medical-funds
Love for the St. Louis	https://www.gofundme.com/32aqwaxw
Donate For Melanie Carriere!	https://www.gofundme.com/jd4atk
Jax's struggle for independence	https://www.gofundme.com/jaxdorward
Assist with a Live Kidney Donation!	https://www.gofundme.com/heathersgiftoflife
Gerry's Kidney Fund!	https://www.gofundme.com/n32vwc
Jason Young's Healing Fund	https://www.gofundme.com/jason-youngs-healing-fund
Kenneth's Renal Transplant Surgery	https://www.gofundme.com/kenneths-renal-transplant-surgery
Help Kindey Donor Brodie Abbs	https://www.gofundme.com/xxgh9h9k
Julie's Animals	https://www.gofundme.com/julies-animals
Kingston's Kidney Cousins	https://www.gofundme.com/KidneyCousins
Support Steve Hay	https://www.gofundme.com/tb9tcr-support-steve
Brianna's Medical and Support Fund	https://www.gofundme.com/briannas-medical-and-support-fund
Kimmie's road to recovery	https://www.gofundme.com/kimmies-road-to-recovery
Audrey's Transplant Fund	https://www.gofundme.com/okgv4s

Title	URL
Let's help our friend Steve Howatt	https://www.gofundme.com/da46s-lets-help-steve
Stephen Crane	https://www.gofundme.com/3sqwn89c
REUBEN'S MEDICAL EXPENSES	https://www.gofundme.com/w8r6kc4
A Sister's Love	https://www.gofundme.com/prunjhwc
Loradel & Darwin Medical Funds	https://www.gofundme.com/Loradel-and-Darwin
Deserving Couple needs our Help!	https://www.gofundme.com/courtneyandpaul
Helping the Craig Family	https://www.gofundme.com/helping-the-craig-family
Jordanne's transplant & recovery	https://www.gofundme.com/x9gfcc
Diamond Rally - Team St. Paul's	https://www.gofundme.com/diamond-rally-team-st-paul-2018
A Kidney for Tiffany	https://www.gofundme.com/usw4n-a-kidney-for-tiffany
Pray for Paul	https://www.gofundme.com/pray-forpaul
Kindness for Kris	https://www.gofundme.com/kindnesskris
Raising money for a kidney	https://www.gofundme.com/e6uabo
Her struggle continues	https://www.gofundme.com/cnqnhzck
Dad's Health	https://www.gofundme.com/24xnubw
Robyn's Emergency	https://www.gofundme.com/Robynneedshelp
Cathie's Recovery	https://www.gofundme.com/u2kgr6p
Cory-Living With Kidney Failure	https://www.gofundme.com/3cqanxc
Laurie Vinson Needs Our Help	https://www.gofundme.com/let-s-help-laurie
Medical expenses	https://www.gofundme.com/medical-expenses-2tph98uc
Support for Johnny's New Kidney :)	https://www.gofundme.com/eu1194
Roach family kidney donation.	https://www.gofundme.com/w3xhb8g
help rebuild pats house so he stays	https://www.gofundme.com/help-pat-keep-above-water
Struggling Cancer Family	https://www.gofundme.com/struggling-cancer-family
My Christmas Wish	https://www.gofundme.com/4vstvv4
Grandma Gets Life Saving Transplant	https://www.gofundme.com/grandma-gets-life-saving-transplant
Dwaynes medical expenses	https://www.gofundme.com/dwaynestransplant
Darryl and Sara's Kidney Fund	https://www.gofundme.com/DarrylSaraKidney
Travel funds for Dialysis 3×week	https://www.gofundme.com/travel-funds-for-dialysis-3xweek
Kidney campaign	https://www.gofundme.com/kidney-campaign
Sheri's Kidney Quality of Life Fund	https://www.gofundme.com/WWWmiracle-Sherry-kidney-donations
Walker's Medical Fund	https://www.gofundme.com/kvutt-walkers-medical-fund
Kidney failure5	https://www.gofundme.com/kidney-failure5
Almie's Kidney Medical Fund	https://www.gofundme.com/almies-kidney-medical-fund
Medical expenses	https://www.gofundme.com/6wbvv7-medical-expenses
Help For Jeremy & Rochelle	https://www.gofundme.com/help-for-jeremy-rochelle
Susie's medical fund	https://www.gofundme.com/hzg24-susies-medical-fund
Brices Medical Fund	https://www.gofundme.com/6fry7v-brices-medical-fund
Alex's transplant recovery	https://www.gofundme.com/healthy-transplant-new-life
Kidney/ Pancreas Costs	https://www.gofundme.com/kidney-pancreas-costs
The Murray Family Crisis	https://www.gofundme.com/the-murray-family-crisis
Help me, help my mom	https://www.gofundme.com/mqvaup-help-me-help-my-mom
Dan's transplant campaign	https://www.gofundme.com/nkb0bs
Nira's Medical Fund	https://www.gofundme.com/niras-medical-fund
Avrie Jaqueline Gauthier	https://www.gofundme.com/r7k6rs
Matts Donation To His Sister	https://www.gofundme.com/matthelpinghissister
New life with transplant costs much	https://www.gofundme.com/new-life-with-transplant-costs-much
Keanna's Medical & Travel Expenses	https://www.gofundme.com/kidneyforkeanna
Two friends one kidney	https://www.gofundme.com/c9oiqw
Christine's Medical Fund	https://www.gofundme.com/8tuew458
Donatinos needed for trip to abilen	https://www.gofundme.com/s8ghvkw
brakes on truck ,,, new glasses	https://www.gofundme.com/my-new-galsses
Chad Penners Medical fund Help!	https://www.gofundme.com/chad-penners-medical-fund-help
Winston's medical expenses	https://www.gofundme.com/2kpadpws
Fund Matt	https://www.gofundme.com/fund-mat
Friend in need	https://www.gofundme.com/ax6qb-friend-in-need
Desiree Bouchard - Kidney Donation	https://www.gofundme.com/desiree-bouchard-kidney-donation
Peter Fox's Kidney Quest	https://www.gofundme.com/oq1po8
Help Stacy Boggs	https://www.gofundme.com/help-stacy-boggs
Family Lopez transplant support	https://www.gofundme.com/family-lopez-transplant-support
A Kidney, for Pete's Sake!	https://www.gofundme.com/akidney4petessake
Needs Double Transplant & Laidoff	https://www.gofundme.com/needs-double-transplant-laidoff
Drielle is Getting a Kidney!	https://www.gofundme.com/engec-drielle039s-getting-a-kidney
Lindsay Villeneuve kidney donor	https://www.gofundme.com/lindsay-villeneuve-kidney-donor
Luke's Kidney donation fund	https://www.gofundme.com/2mdaegc
Help Shemim through her Transplant!	https://www.gofundme.com/2tw7q57w
Christopher's Kidney Fund	https://www.gofundme.com/2jx2ytfw
Florence and Terence's transplant	https://www.gofundme.com/zbxzjg3j
Get Amber A Kidney	https://www.gofundme.com/pf637k
Father saves Daughter with Kidney	https://www.gofundme.com/helpjolinaskidney
transplant for Jessica Peterson	https://www.gofundme.com/fundsfortransplant
Cory's battle with kidney disease	https://www.gofundme.com/corys-battle-with-kidney-disease
AL's kidney fund	https://www.gofundme.com/y6v4vq7f
Gwennael's Transplant Journey	https://www.gofundme.com/gwennael
Natalie's Transplant Fund	https://www.gofundme.com/NatsTransplantFund
Maureen and Carl's Kidney Campaign	https://www.gofundme.com/maureen-and-carls-kidney-campaign
Find my Mom a kidney please	https://www.gofundme.com/find-my-mom-a-kidney-please
Need extra cash for kidney	https://www.gofundme.com/need-extra-cash-for-kidney
Help Shelley get a transplant	https://www.gofundme.com/help-shelley-get-a-transplant
I'm donating my kidney	https://www.gofundme.com/im-donating-my-kidney
Jermaine's coming Artificial Kidney	https://www.gofundme.com/jermaines-coming-artificial-kidney
Hewlette Marielle's Medical Funds	https://www.gofundme.com/ne6zpe-hewlette-marielles-medical-funds
Peter's Fund	https://www.gofundme.com/petercameron
Our Fight for Kris and His Family	https://www.gofundme.com/our-fight-for-kris-and-his-family
Go Fund the kay's	https://www.gofundme.com/ixn33k
Surgery a success!!	https://www.gofundme.com/t6y84xvg
Dad's Medical Fund	https://www.gofundme.com/no21qc
Ken's Medical Fund	https://www.gofundme.com/ms-kens-medical-fund
Miller/Clark Medical Fund	https://www.gofundme.com/millerclark-medical-fund
Shon's Drive To Dialysis	https://www.gofundme.com/pez1go
Helping Dexter's Dad	https://www.gofundme.com/supportforeinar
Shane's Fundraiser	https://www.gofundme.com/kul3d8
MacIntyre Family Fund	https://www.gofundme.com/2bub3zwc
Help With Medical expenses	https://www.gofundme.com/29x2nx6v
Devon's New Life	https://www.gofundme.com/2am3h9vg
Carol Pollard's dialysis & medical	https://www.gofundme.com/2k9weje4
My sister, saved my sons life	https://www.gofundme.com/3zehwzhj
Donations for sonya wilson	https://www.gofundme.com/bjvkrh78
Help Kyle	https://www.gofundme.com/help-kyle-2vs87pd7
Medical help	https://www.gofundme.com/hsd7jy6k
Angela's Healing	https://www.gofundme.com/support-for-angelas-healing
Help Save Erica	https://www.gofundme.com/savejamie2017
Leah Kidney donor for Dallas	https://www.gofundme.com/leah-kidney-donor-for-dallas
Bonnie's road to recovery	https://www.gofundme.com/bonnies-road-to-recovery
Mother of two donating kidney	https://www.gofundme.com/momdonateskidney
Local Mom to give kidney to another	https://www.gofundme.com/ol5tj8
Colleen Keefe Medical Fundraiser	https://www.gofundme.com/colleenkeefe
Brads Portable Oxygen Concentrator	https://www.gofundme.com/bradsoxygenconcentrator
Farah's life line	https://www.gofundme.com/farahs-life-line
Chryssie's New Kidney Fund	https://www.gofundme.com/chryssies-new-kidney-fund
Donor trip	https://www.gofundme.com/donor-trip
I'm Donating a Kidney!	https://www.gofundme.com/baf4m0
My Little Boy Needs a Kidney	https://www.gofundme.com/my-little-boy-needs-a-kidney
Sheldon's Kidney Medical Fund	https://www.gofundme.com/g23fss3s
help Bennett find a kidney	https://www.gofundme.com/dyrb04
Karin needs a kidney	https://www.gofundme.com/karin-needs-a-kidney
Sheap Family Relief Fund	https://www.gofundme.com/sheap-family-relief-fund
Help Baby Sophia Recover!!!	https://www.gofundme.com/kstfuo
Jeff's Medical Fund	https://www.gofundme.com/jeffs-medical-fund
Kristen's Kidney Donation Fund	https://www.gofundme.com/kristens-kidney-donation-fund
Need help while on kidney Dialysis	https://www.gofundme.com/need-help-while-on-kidney-dialysis
Jay's Medical Fund	https://www.gofundme.com/j6h83-jays-medical-fund
Annie's Fundraising Campaign	https://www.gofundme.com/og6hn0
